# Biomechanical evaluation of a new intramedullary nail compared with proximal femoral nail antirotation and InterTAN for the management of femoral intertrochanteric fractures

**DOI:** 10.3389/fbioe.2024.1353677

**Published:** 2024-02-23

**Authors:** ChaoFeng Wang, Ning Duan, Zhong Li, Teng Ma, Kun Zhang, Qian Wang, Qiang Huang

**Affiliations:** Department of Orthopedics, Hong Hui Hospital, Xi’an Jiaotong University, Xi’an, Shaanxi, China

**Keywords:** mechanical, femur, intertrochanteric fracture, stability, finite element method, implant

## Abstract

**Purpose:** Surgical treatment is the main treatment method for femoral intertrochanteric fractures (FIFs), however, there are lots of implant-related complications after surgery. Our team designed a new intramedullary nail (NIN) to manage such fractures. The purpose of this study was to introduce this new implant and compare it with proximal femoral nail antirotation (PFNA) and InterTAN for treating FIFs.

**Methods:** An AO/OTA 31-A1.3 FIF model was built and three fixation models were created via finite element method, comprising PFNA, InterTAN, and the NIN. Vertical, anteroposterior (A-P) bending, and torsional loads were simulated and applied to the three fixation models. Displacement and stress distribution were monitored. In order to compare PFNA and the NIN deeply, finite element testing was repeated for five times in vertical load case.

**Results:** The finite element analysis (FEA) data indicated that the NIN possessed the most outstanding mechanical properties among the three fixation models. The NIN model had lower maximal stress at implants compared to PFNA and InterTAN models under three load conditions. The trend of maximal stress at bones was similar to that of maximal stress at implants. Besides, the NIN model showed smaller maximal displacement compared with PFNA and InterTAN models under vertical, A-P bending, and torsional load cases. The trend for maximal displacement of fracture surface (MDFS) was almost identical with that of maximal displacement. In addition, there was significant difference between the PFNA and NIN groups in vertical load case (*p* < 0.05).

**Conclusion:** Compared with PFNA and InterTAN, the NIN displayed the best mechanical properties for managing FIFs, including the lowest von Mises stress at implants and bones, and the smallest maximal displacement and MDFS under vertical, A-P bending, and torsional load cases. Therefore, this study might provide a new choice for patients with FIFs.

## Introduction

Hip fractures are a common type of fractures for the elderly. These fractures are usually osteoporotic fractures which are caused by low-energy damage (Chen et al., 2022). It is estimated that the annual cases are about 1.6 million worldwide (Huette et al., 2020). Femoral intertrochanteric fractures (FIFs) refer to a fracture in the area between the greater and lesser trochanter (Huang et al., 2022). It accounts for approximately half of total cases of hip fractures. Due to the intensification of aging population, the number of patients with FIFs continues to increase, and the medical, economic, and social burden it brings becomes heavier. Timely surgical management is recommended for such patients to decrease the risk of bed-rest related complications (Kokoroghiannis et al., 2012). Yet, even those undergoing surgeries, the mortality rate within 1 year after surgery can reach 6.6%–36.4% (Chlebeck et al., 2019). Hence, how to better treat such patients is a challenge faced by trauma surgeons.

Currently, intramedullary nails are the first choice for the treatment of FIFs as intramedullary fixation is a central and minimally invasive fixation method (Queally et al., 2014; Brox et al., 2015). The commonly used implants for fixing FIFs can be divided into single-screw and dual-screw cephalomedullary nails (Yang et al., 2023). The single-screw cephalomedullary nails include PFNA, Gamma3 nail, etc. Compared to Gamma3 nails, the PFNA is more widely used for treating FIFs. The cephalomedullary screw of PFNA is a spiral blade, which can compress cancellous bones, and increase the contact area with the cancellous bones (Radaideh et al., 2018). During the insertion of the spiral blade, additional hole expansion is not required. This could add extra anchoring, which is especially suitable for patients with osteoporosis. Even though patients with FIFs have been successfully treated by the PFNA and Gamma3 nails, insufficient antirotation of the proximal fragment, migration of the cephalomedullary screw, and cut-out were frequently reported (Domingo et al., 2001; Al-yassari et al., 2002). Then, the U-blade Gamma nail was created to enhance the biomechanical properties of femoral intertrochanteric fractures. The U-blade lag screw could enlarge the contact area between the screw and cancellous bones by a U-shape clip, thus increasing antirotation and the integrated stability (Lenich et al., 2010). However, more literature had a negative point towards the U-blade, which may be due to the prolonged surgical time, increased costs, and no significant reduction in complications (Lang et al., 2016; Lang et al., 2019). The dual-screw cephalomedullary nails contain proximal femoral nail (PFN), InterTAN, etc. Compared to extramedullary implants, the PFN has been proven to have advantages in the treatment of FIFs. Yet, the incidence of postoperative complications such as Z-effect, reverse Z-effect, screw cut-out, and inversion collapse could be as high as 31% (Hohendorff et al., 2005). InterTAN is also composed of two cephalomedullary screws that allows for axial compression, provides extra resistance to the proximal fragment rotation, and decreases the incidence of Z-effect (Hoffmann et al., 2013). However, the two cephalomedullary screws of InterTAN are arranged tightly parallel to each other, approximating “one screw” with a larger diameter. Its resistance on the rotation of the femoral head is ultimately limited.

**FIGURE 1:**
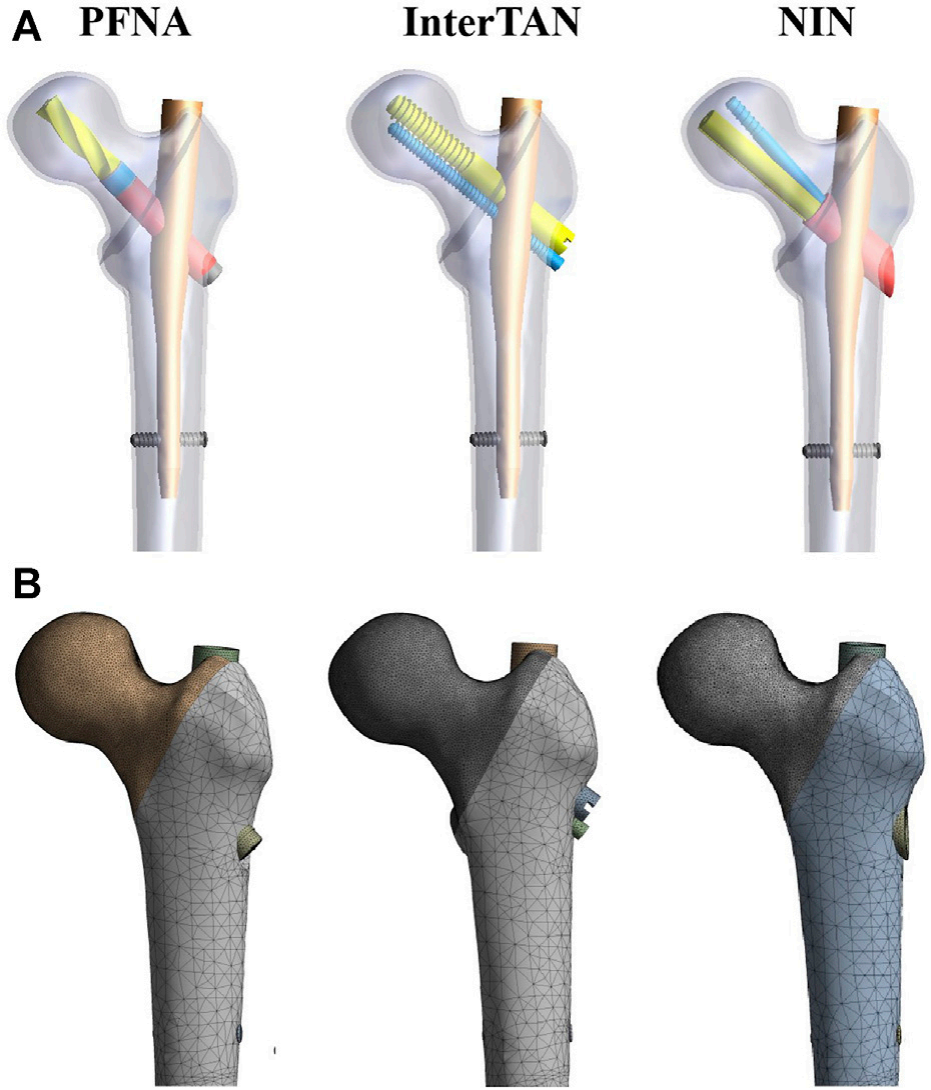
Three fixation models and mesh figures. **(A)** The three fixation models when finite element models were assembled. The models included PFNA, InterTAN, and the NIN. **(B)** Mesh figures of three fixation models. PFNA stands for proximal femoral nail antirotation. NIN stands for the new intramedullary nail.

A reasonable design of cephalomedullary nails is crucial for reducing stress concentration and improving fixation stability (Chantarapanich and Riansuwan, 2022). Based on the current situations, the authors designed a new intramedullary nail to improve the treatment of FIFs (Figure 1A). The proximal part of the NIN consisted of two cephalomedullary screws and one sleeve. The lower cephalomedullary screw fixs the fragment while the upper screw provides sliding compression and antirotation. Two cephalomedullary screws are arranged at an acute angle to avoid the Z-effect. The two cephalomedullary screws of the NIN, as a whole, can slide slightly inside the sleeve to further compress the fracture site of FIFs. In previous research, we have demonstrated that this new intramedullary nail could provide good biomechanical stability for the treatment of femoral neck fractures (Huang et al., 2023). Yet, it is unclear whether the NIN could provide a good fixation for patients with femoral intertrochanteric fractures. In this study, we constructed an AO/OTA 31-A1.3 FIF model. Three fixation models were created via finite element method, comprising PFNA, InterTAN, and the NIN. Finite element analysis is a virtual simulation method that utilizes mathematical modeling. It can reflect stress distribution and displacement changes by applying loads virtually, thereby evaluating the stability of orthopedic implants (Jitprapaikulsarn et al., 2021). We simulated vertical, A-P bending, and torsional loads in this research. The corresponding stress distribution and displacement change were analyzed. Moreover, in order to compare PFNA and the NIN deeply, finite element testing was repeated for five times in vertical load case. We supposed that compared to PFNA and InterTAN, the NIN had better biomechanical properties.

## Materials and methods

### 3D model construction for the femur and implants

A healthy male volunteer (70 years old, 68 Kg) without any history of hip or systemic diseases was recruited. A 3D femur model was created on the basis of CT scan data of his left leg via Mimics (Materialise, Leuven, Belgium). Voltage operating scope was set as 70–140 kV while current for 30–800 mA during CT scanning. Cortical and cancellous bones were identified via Hounsfield Unit (HU) and the boundary was set as 700 (Abdul Wahab et al., 2020). AO/OTA 31-A1.3 is a type of FIFs with a high incidence (Catania et al., 2019; Shi et al., 2021). An AO/OTA 31-A1.3 FIF model was constructed through an osteotomy line from the greater trochanter to the lesser trochanter according to previous literature (Meinberg et al., 2018; Wang et al., 2022). Computer-aided design software (Dassault Systemes SolidWorks Corp., United States) was used to build three implant models, including PFNA, InterTAN, and the NIN models. Then, the three implant configurations were converted to the stereolithography format and imported into the 3-Matic (Materialise, Leuven, Belgium). In 3-Matic software, the above implant models were assembled onto the FIF models, respectively. Three fixation models were shown schematically in Figure 1A. The parameters for the NIN were as follows. The length of the NIN is 170 mm. The diameter of the proximal and distal main nail is 17 mm and 10 mm, respectively. The diameter for the two cephalomedullary screws, and the sleeve is designed as 10 mm, 6.4 mm, and 14 mm, respectively. Besides, the included angle between the lower cephalomedullary screw and the main nail is set as 130°. The angle is designed as 7.5° between the two cephalomedullary screws.

### Settings for finite element analysis

**FIGURE 2:**
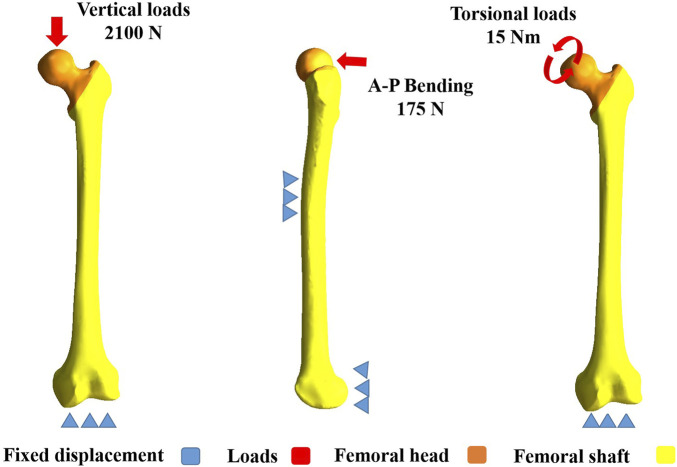
Boundary conditions of vertical, A-P bending, and torsional loads. For vertical loads, it was set as 2,100 N, applying axially to the femoral head. For A-P bending, it was set as 175 N, applying laterally to the femoral head. For torsional loads, it was set as 15 Nm, applying to the femoral head along the femoral neck axis. A-P stands for anteroposterior.

Three fixation models were set as homogeneous, linear, and isotropic material properties. Tetrahedral elements were used for meshing in finite element settings. In order to assess the reliability of three fixation models, a convergence study was conducted according to previous literature (Huang et al., 2023). With respect to maximal Degree of Freedom, the field indexes of the two types of elements, containing strain energy and displacement, were within the scope of 5%. Moreover, there was no maximal stress point. Elements and nodes of three fixation models were illustrated in Table 1. Mesh figures of three fixation models were shown in Figure 1B. All three implants were set to Titanium alloy due to its superior material characteristics, such as perfect biocompatibility, and good corrosion resistance (Meng et al., 2022). The elastic modulus was set as 16,800 MPa for cortex, 840 MPa for cancellous bones, and 110,000 MPa for Titanium alloy based on similar studies (Li et al., 2018; Huang et al., 2023). The value of Poisson’s ratio was assumed to be 0.3 for cortical bones and Titanium alloy while 0.2 for cancellous bones, respectively (Li et al., 2018). All contact surfaces were supposed to friction contact, such as the contact between bones and implants, while the friction coefficient was assumed to be 0.4 (Viceconti et al., 2000). Boundary conditions for vertical, A-P bending, and torsional loads were illustrated schematically in Figure 2. The vertical loads were set as 2,100 N, applying vertically on the top of the femoral head (Zhang et al., 2011; Li et al., 2019). The distal femur was strictly fixed to forbid micro movement of models during testing. For A-P bending, the mid and distal femur was tightly fixed. The A-P bending loads were adjusted to 175 N, applying laterally to the femoral head surface (Li et al., 2018). Notably, during the process of torsional testing, a load of 15 Nm was applied along the femoral neck axis according to previous studies (Li et al., 2018).

**TABLE 1:** Number of nodes and elements for PFNA, InterTAN, and the NIN models.

Model	Nodes	Elements
PFNA	951701	634942
InterTAN	987494	649750
NIN	943859	625403

PFNA, proximal femoral nail antirotation; NIN, the new intramedullary nail.

### Evaluation indicators

Maximal displacement, maximal displacement of fracture surface (MDFS) and von Mises stress at implants and bones were evaluated under vertical, A-P bending, and torsional loads. The PFNA model was assumed to be the control group due to its widespread application in recent years and was considered to have good biomechanical stability. The variation rate (VR) was calculated according to the following arithmetic formula: VR =(V_1_ − Vn)/V_1_×100%. Vn represents the value of InterTAN, or the NIN models. V_1_ represents the value of the PFNA model. Moreover, in order to compare PFNA and the NIN deeply, finite element testing was repeated for five times in vertical load case.

### Statistical analysis

SPSS 23.0 software was applied to conduct statistical analysis. For the PFNA and NIN models, the values of displacement and von Mises stress in vertical load case were compared by the Student’s t-test. *P* < 0.05 was considered to be statistically different.

## Results

### Von Mises stress at implants

**FIGURE 3:**
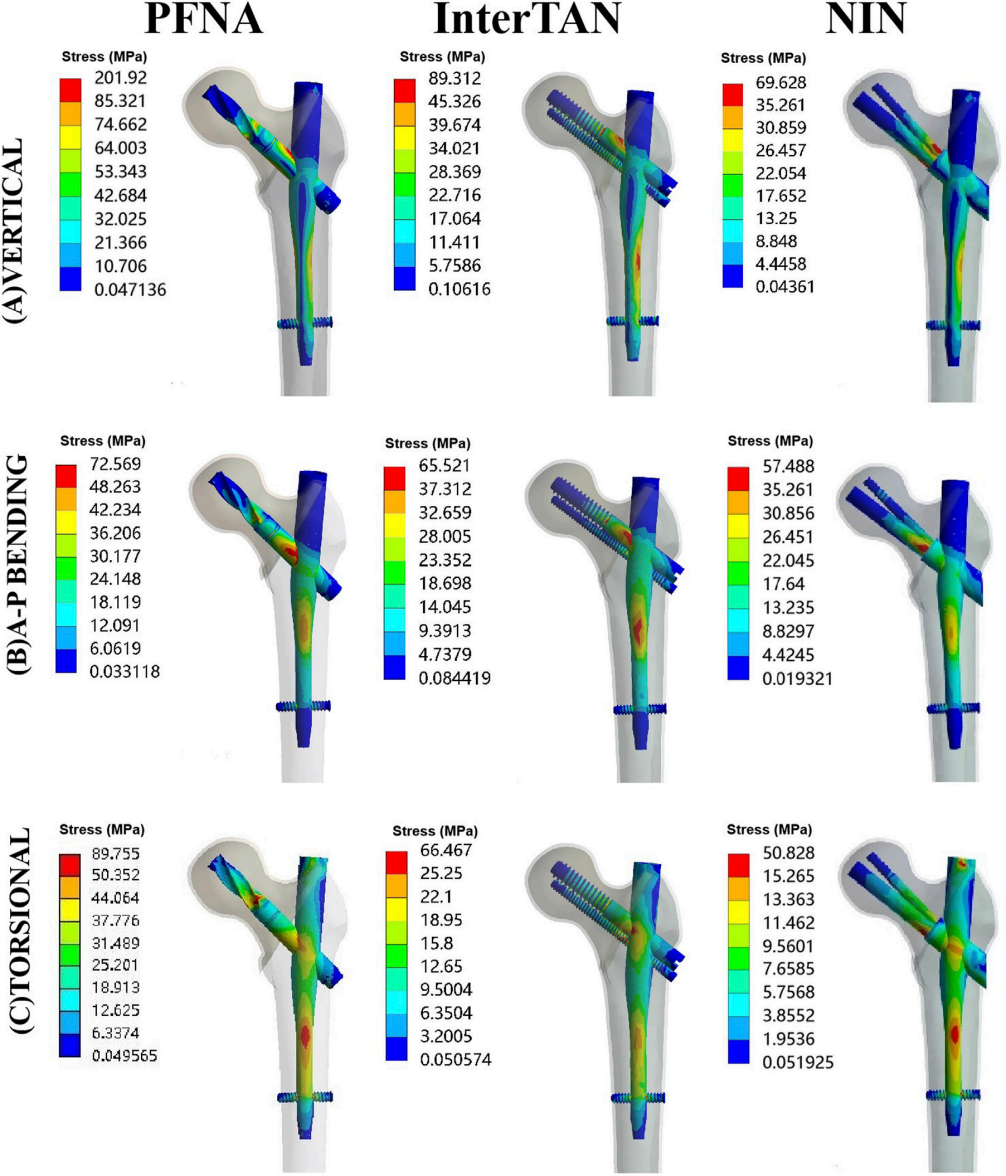
Contour images of von Mises stress at implants for three fixation models under vertical, A-P bending, and torsional loads. The models included PFNA, InterTAN, and the NIN. A-P stands for anteroposterior. PFNA stands for proximal femoral nail antirotation. NIN stands for the new intramedullary nail.

Contour images of Von Mises stress at implants for three fixation models under vertical, A-P bending, and torsional loads were illustrated schematically in Figure 3. The stress concentration area and maximum stress point for three implants were almost all located at the intersection between the main nail and the cephalomedullary screw. Under vertical loads of 2100 N, in order of the highest to the lowest maximal stress at implants, the three fixation models were ranked as follows: PFNA, InterTAN, and the NIN. When A-P bending and torsional loads were applied, they were also ranked as follows: PFNA, InterTAN, and the NIN. The maximal stress reduction rate of the NIN model relative to the PFNA model was 65.5% under vertical loads, 20.8% under A-P bending loads, and 43.4% under torsional loads, respectively.

### Von Mises stress at bones

**FIGURE 4:**
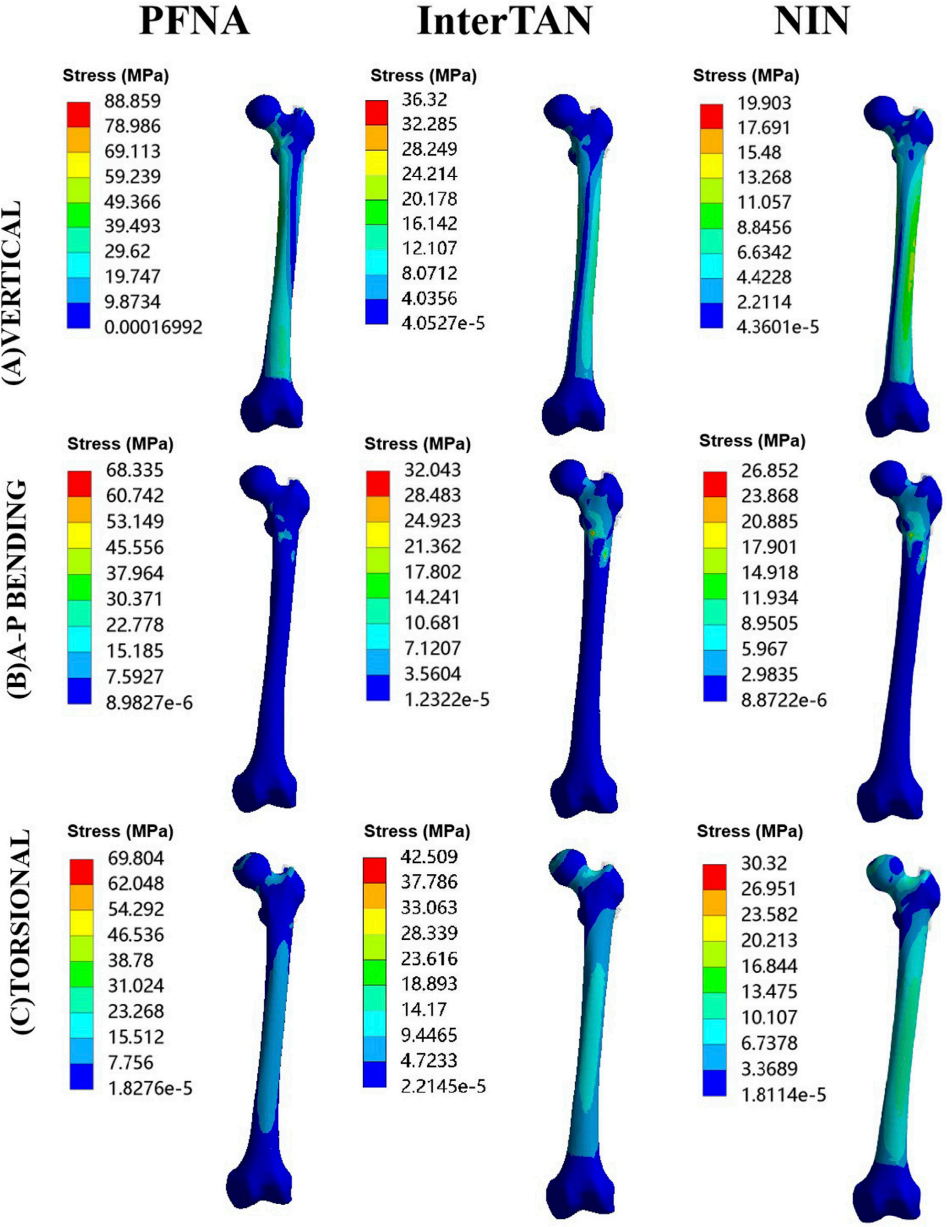
Contour images of von Mises stress at bones for three fixation models under three load conditions. The models included PFNA, InterTAN, and the NIN. PFNA stands for proximal femoral nail antirotation. NIN stands for the new intramedullary nail.

Contour images of Von Mises stress at bones under three load cases were illustrated schematically in Figure 4. Under vertical load conditions, the maximal stress at bones for PFNA, InterTAN and the NIN was 88.86 MPa, 36.32 MPa, and 19.90 MPa, respectively. Compared with the PFNA model, the reduction rate of maximal stress at bones for the NIN model was 77.6% under vertical loads. Moreover, the value of maximal stress at bones was 68.34 MPa, 32.04 MPa, and 26.85 MPa under A-P bending loads while 69.80 MPa, 42.51 MPa, and 30.32 MPa under torsional loads for PFNA, InterTAN and NIN models. Compared to the PFNA model, the reduction rate for the NIN model was 60.7% under A-P bending loads while 56.6% under torsional loads, respectively.

### Maximal displacement of three different models

**FIGURE 5:**
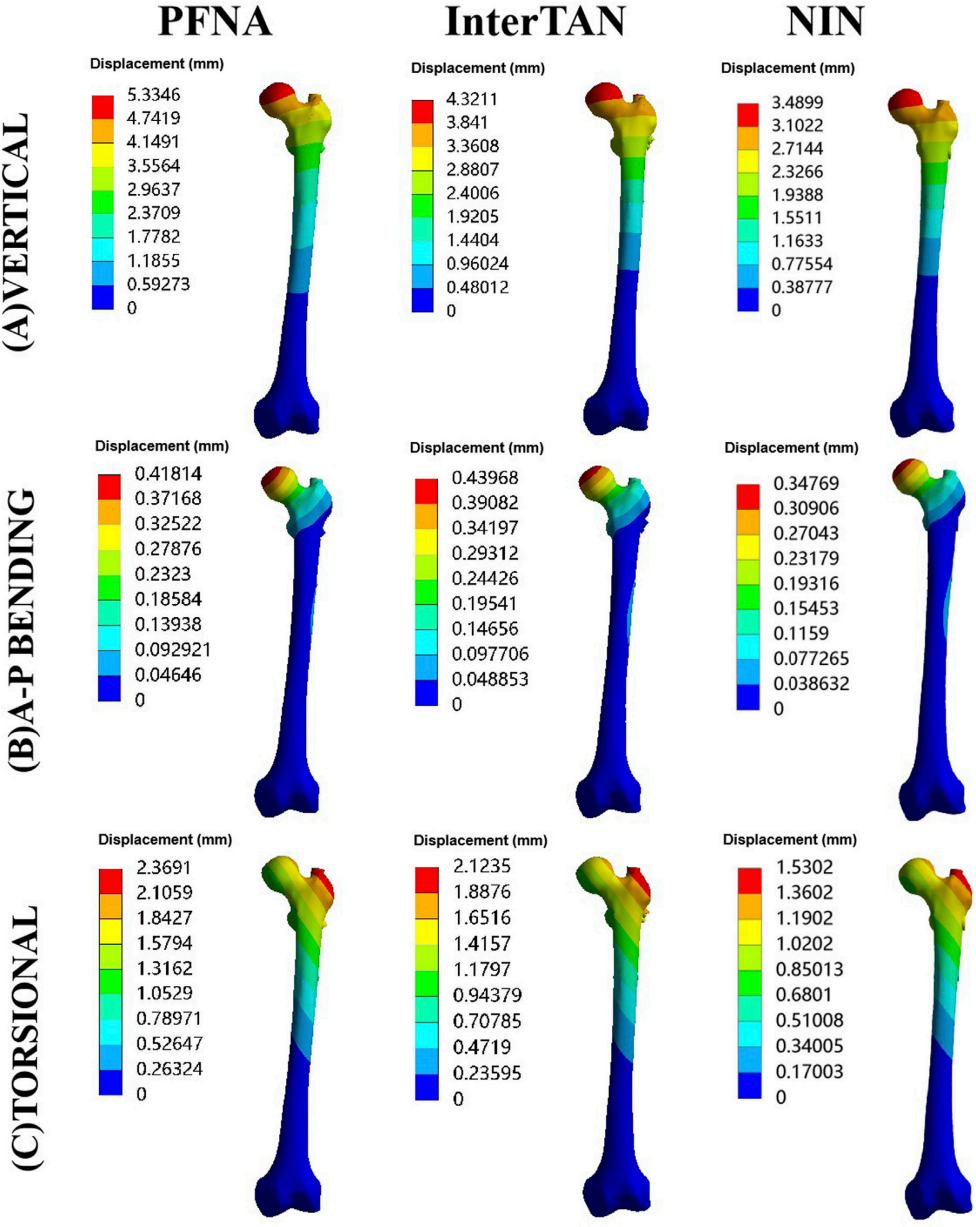
Contour pictures of maximal displacement for three fixation models under three load conditions. The models included PFNA, InterTAN, and the NIN. PFNA stands for proximal femoral nail antirotation. NIN stands for the new intramedullary nail.

Contour pictures of maximal displacement under vertical, A-P bending, and torsional loads were exhibited in Figure 5. Under vertical load conditions, in order of the largest to the smallest value of this indicator, the three fixation models were ranked as follows: PFNA, InterTAN, and the NIN. When A-P bending loads were applied, these models were ranked as follows: InterTAN, PFNA and the NIN. Besides, for torsional testing, they were ranked as follows: PFNA, InterTAN, and the NIN. The values of maximal stress for the NIN model were less than those of the PFNA model under three different load conditions. The reduction rate of this indicator for the NIN model relative to the PFNA model was 34.6%, 16.8%, and 35.4% under vertical, A-P bending, and torsional loads, respectively.

### Maximal displacement of fracture surface (MDFS) for three fixation models

**FIGURE 6:**
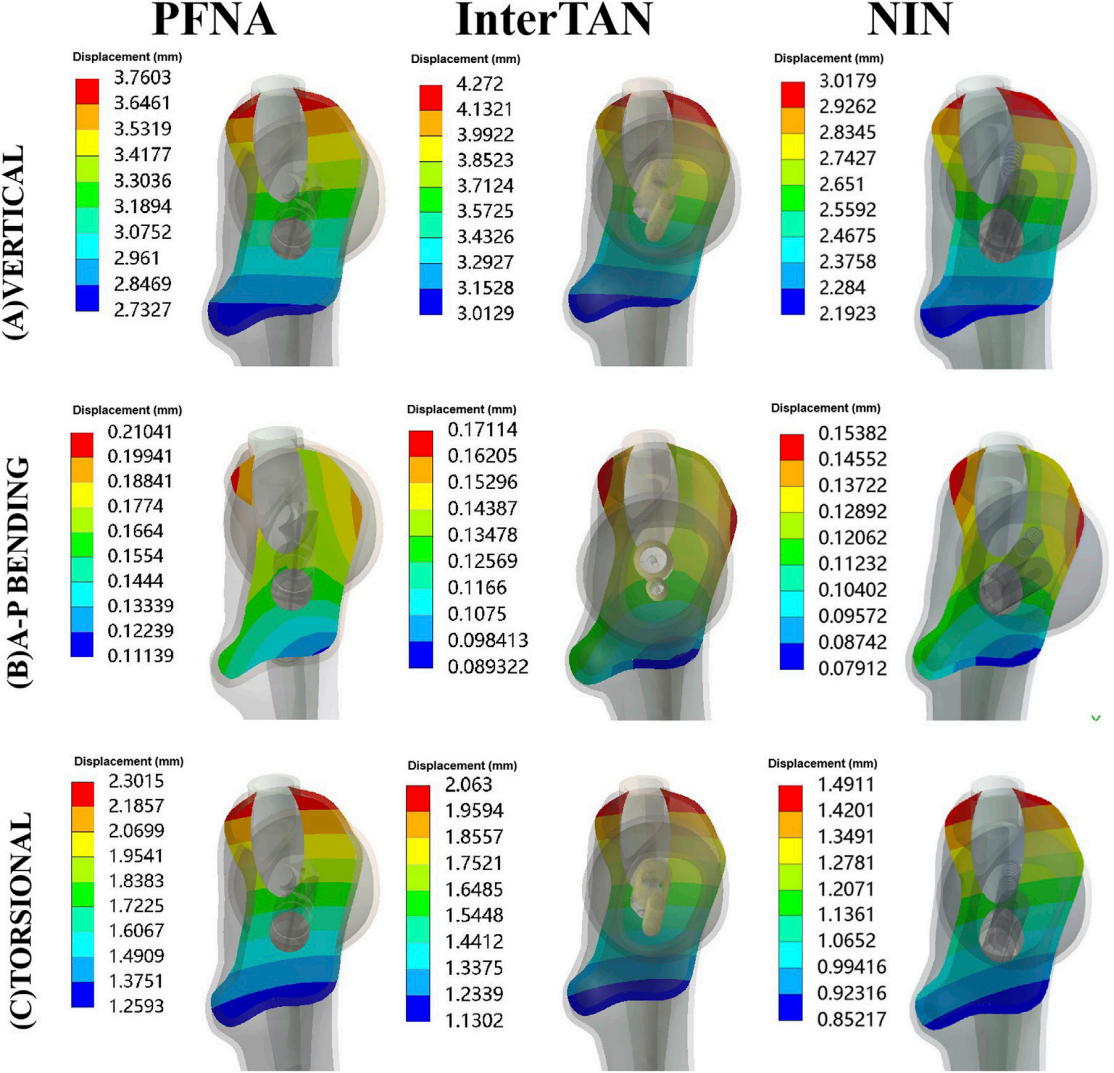
Contour pictures of maximal displacement of fracture surface for three fixation models under three load conditions. The models included PFNA, InterTAN, and the NIN. PFNA stands for proximal femoral nail antirotation. NIN stands for the new intramedullary nail.

Contour pictures of MDFS for three fixation models under vertical, A-P bending, and torsional loads were displayed in Figure 6. Specifically, under vertical loads of 2100 N, the values of MDFS for PFNA, InterTAN and NIN models were 3.76 mm, 4.27 mm, and 3.02 mm, respectively. They were 0.21 mm, 0.17 mm, and 0.15 mm for A-P bending testing while 2.30 mm, 2.06 mm, and 1.49 mm for torsional experiment, respectively. Compared to the PFNA model, the reduction rate of MDFS for the NIN model was 19.7% under vertical loads (26.9% for A-P bending loads and 35.2% for torsional loads). The values of MDFS for the NIN model were lower than those of the PFNA model under three load cases.

### Statistical analysis under vertical load case

Statistical analysis data were exhibited in Table 2 for the PFNA and NIN models under vertical loads of 2100 N. The mean values of maximal stress at implants were 203.15 ± 22.68 MPa for the PFNA models and 68.79 ± 15.18 MPa for the NIN models, and the difference was statistically significant (*p* < 0.05). The mean values of maximal stress at bones were 85.63 ± 14.22 MPa for the PFNA models and 20.54 ± 3.69 MPa for the NIN models, with significant difference between the two fixation models (*p* < 0.05). The mean values of maximal displacement were 5.18 ± 0.27 mm and 3.51 ± 0.16 mm for the PFNA and NIN models, and the difference was statistically significant (*p* < 0.05). The mean values of MDFS were 3.65 ± 0.32 mm for the PFNA models and 2.99 ± 0.24 mm for the NIN models, and there was significant difference between the two fixation models (*p* < 0.05). Hence, in vertical load case, the NIN model displayed better mechanical stability than the PFNA model for fixing femoral intertrochanteric fractures.

**TABLE 2:** Comparison of mechanical stability between the PFNA and NIN in vertical load case.

Mechanical parameters	PFNA	NIN	t	*p*
Maximal stress at implants (MPa)	203.15 ± 22.68	68.79 ± 15.18	11.01	0.000
Maximal stress at bones (MPa)	85.63 ± 14.22	20.54 ± 3.69	9.91	0.001
Maximal displacement (mm)	5.18 ± 0.27	3.51 ± 0.16	11.90	0.000
Maximal displacement of fracture surface (mm)	3.65 ± 0.32	2.99 ± 0.24	3.69	0.008

Notes: PFNA, stands for proximal femoral nail antirotation; NIN, stands for the new intramedullary nail.

## Discussion

Our results indicated that compared with PFNA and InterTAN, the new intramedullary nail had more uniform stress distribution and better biomechanical stability under vertical, A-P bending, and torsional loads. Moreover, the dual-screw implants (InterTAN and the NIN) had better mechanical properties than the single-screw implant (PFNA). Two cephalomedullary screws arranged at an acute angle (the NIN) had better biomechanical stability than that of tightly parallel arrangement (InterTAN).

Scholars have reached a consensus that intramedullary fixation is superior to extramedullary fixation for treating FIFs, due to the shorter level arm and central fixation characteristics (Haidukewych, 2009; Parker and Handoll, 2010). However, there is still significant controversy over whether to use dual-screw or single-screw intramedullary implants to fix FIFs. In earlier years, surgeons used PFN to fix intertrochanteric fractures. The two cephalomedullary screws of PFN were arranged in parallel and evenly distributed in the upper and lower one-third of the femoral head. Yet, the Z-effect was commonly observed in FIF patients, resulting in implant failure and a high re-operation rate (Hohendorff et al., 2005). For the elderly, this complication caused greater harm. Based on this, trauma surgeons turned their attention to PFNA and Gamma nails. It was reported that patients using PFNA or Gamma3 nails achieved similar clinical effects for treating FIFs (Bonnaire et al., 2020). Many studies have compared PFN and PFNA from different perspectives and demonstrated that the PFNA was superior to PFN in treating FIFs (Mallya et al., 2019; Baek et al., 2020). Although the PFNA is currently the most widely used intramedullary implant, reports on its complications are not uncommon (Queally et al., 2014; Radaideh et al., 2018; Lang et al., 2019). With the emergence of InterTAN nail, scholars have once again developed expectations for the dual-screw configuration. Some clinical studies have shown that compared to PFNA, insertion of InterTAN could significantly reduce complications in patients with trochanteric fractures (Nherera et al., 2018; Date et al., 2020). Biomechanical experiments showed that InterTAN had better mechanical stability than the PFNA device (Santoni et al., 2016). Our results by this study also confirmed the above points via finite element method. Nevertheless, the dual-screw structure of InterTAN also has the following shortcomings, such as limited antirotation and stress concentration of two cephalomedullary screws. The tight and parallel design of the two cephalomedullary screws for InterTAN may be the cause of the above problems.

To improve the treatment of FIFs, we developed the new intramedullary nail. According to previous research, the loads acting onto the femoral head are as high as three times of one’s body weight during walking (Bergmann et al., 2001). For a patient with a weight of 60–70 kg, the maximal loads applying to the femoral head surface are approximately 2100 N during walking. Therefore, the vertical loads were set as 2100 N in this study. The use of von Mises stress can explain the stress that the bone/implant is subjected to locally. We evaluated the biomechanical stability of three different implants through stress and displacement under the same boundary conditions. The smaller the displacement value is, the more stable the fixation model is. The von Mises stress reflects whether the implant is easy to break and the stress distribution at different positions/connections. The greater the stress is, the easier the implant is to break.

Our finite element data indicated that the NIN displayed the best mechanical properties among the three implants (PFNA, InterTAN, and the NIN). In addition, when finite element testing was repeated for five times in vertical load case, the values of von Mises stress and displacement were significant different between the PFNA and NIN fixation models (*p* < 0.05). The unique mechanical structure of the NIN may result in these results. The NIN provided good antirotation, possibly due to the useful design of its proximal part. Indeed, as exhibited in the data under torsional load conditions, the NIN had smaller displacement and more uniform stress distribution than the single-screw implant of PFNA and the dual-screw InterTAN whose cephalomedullary screws were in parallel and tight arrangement. The NIN dispersed stress and avoided the Z-effect, possibly due to the acute angle structure and interlocking of the two cephalomedullary screws. In all three fixation models of our study, the intersection area between the cephalomedullary screw and the main nail was a stress concentrated region. The maximal stress of the NIN model was lower than that of PFNA and InterTAN under three load conditions. This may also be due to the effective design of proximal cephalomedullary screws of the NIN, which were evenly distributed in the femoral head to a certain extent. Actually, our finite element data indicated that the cephalomedullary screw design of the NIN was beneficial for stress dispersion. Wang et al. introduced the proximal femur bionic nail (PFBN) for fixing FIFs (Wang et al., 2022). Two neck screws of PFBN intersect at an acute angle in the femoral head. Based on Wang’s study, the PFBN showed better mechanical stability compared to PFNA and InterTAN under axial loads of 2100 N (Wang et al., 2022). For the design of the NIN, there are some similarities with that of PFBN. They both belong to dual-screw structures, intersecting at an acute angle. However, the two cephalomedullary screws of PFBN are statically stable. On the contrary, the two neck screws of the NIN, as a whole, can slide slightly inside the sleeve to further compress the fracture site of FIFs. This is beneficial for eliminating fracture gaps and promoting the healing of intertrochanteric fractures. The femoral neck system (FNS) used for treating femoral neck fractures is also designed based on the interlocking of two cephalomedullary screws and sliding compression (Davidson et al., 2022). The structure of two cephalomedullary screws for the NIN is similar to that of the FNS in a way. Yet, the screw-plate structure of the FNS essentially belongs to extramedullary fixation. The FNS has a long lever arm, which is prone to stress concentration. Unlike the FNS, the NIN belongs to intramedullary fixation with a short moment. When the femoral head is subjected to different loads, stress can be transmitted to the NIN and then the femoral medullary cavity via a short lever arm. In this situation, stress is easily dispersed. This will reduce the risk of implant failure and finally improve the treatment of FIFs.

This study still has some limitations. Firstly, the finite element analysis method was used to identify the biomechanical stability of PFNA, InterTAN, and the NIN for fixing FIFs under vertical, A-P bending, and torsional loads. No model validation was conducted in this study, which explicitly was a common phenomenon of similar simulation researches. However, unlike the actual values of cadaver bones, the authors tried to make comparisons among three implant models based on the same femur under the same loads and boundary conditions. Hence, the lack of model validation might be reasonable in a way. Sometimes cadaver bones do not provide reliable results due to variation in quality. Secondly, ligaments, muscles, tendons, etc., were ignored during 3D model construction. We focused on the impact of different implants on FIF, so it might be necessary to ignore the influence of soft tissues. This was beneficial to ensure that a single variable was included in the study. Thirdly, during finite element settings, isotropic, linear, and homogenous material properties were assumed for femurs. Yet, real cortical and cancellous bones possess heterogeneous properties. To reduce additional time consumption during model construction and given that heterogeneous models could not be established via the current computer simulation technique, therefore isotropic and homogeneous material properties were set in this study. Notably, we will conduct further research on the NIN in future studies.

## Conclusion

Compared with PFNA and InterTAN, the new intramedullary nail displayed the best mechanical properties for managing femoral intertrochanteric fractures, including the lowest von Mises stress at implants and bones, and the smallest maximal displacement and MDFS under vertical, A-P bending, and torsional load cases. Therefore, this study might provide a new choice for patients with femoral intertrochanteric fractures.

## Data Availability

The original contributions presented in the study are included in the article/Supplementary material, further inquiries can be directed to the corresponding authors.
